# A prospective, open-label feasibility study protocol of home-based transcranial direct current stimulation for major depressive disorder in elective lumbar spine surgery candidates

**DOI:** 10.3389/fnhum.2026.1860063

**Published:** 2026-06-11

**Authors:** Arjun K. Menta, Samuel P. Bronckers, Fernando S. Goes, Timothy F. Witham, David B. Cohen, Amit Jain, Sang H. Lee, Lee H. Riley, Khaled M. Kebaish, Daniel Lubelski, Ali Bydon, Nicholas Theodore, Tej D. Azad

**Affiliations:** 1 Department of Neurosurgery, Johns Hopkins University School of Medicine, Baltimore, MD, United States; 2 Department of Psychiatry, Johns Hopkins University School of Medicine, Baltimore, MD, United States; 3 Department of Orthopaedic Surgery, Johns Hopkins University School of Medicine, Baltimore, MD, United States; 4 Department of Neurosurgery, University of Arizona-Phoenix, Phoenix, AZ, United States

**Keywords:** depression, neuromodulation, prehabilitation, spine surgery, tDCS

## Abstract

**Background:**

Depression is common among elective lumbar spine surgery candidates and is associated with worse postoperative outcomes. Home-based transcranial direct current stimulation (tDCS) has demonstrated safety and feasibility, with evidence of antidepressant effects in remote randomized and open-label studies of major depressive disorder (MDD). Whether this approach can be feasibly and acceptably implemented as a preoperative intervention in lumbar spine surgical populations is unknown.

**Methods:**

We propose a prospective, open-label, single-arm feasibility study evaluating preoperative, home-based tDCS for elective, non-revision lumbar spine surgery candidates with MDD. Adults aged 22–75 years with DSM-5 MDD and at least moderate depressive symptom severity [self-reported Montgomery–Åsberg Depression Rating Scale (MADRS-S) ≥ 20] being evaluated for elective lumbar spine surgery will receive home-based tDCS. Key exclusion criteria include history of seizure disorder, implanted cranial electronic or metallic devices, clinically unstable psychiatric conditions, prior brain stimulation therapy, and use of medications that significantly affect cortical excitability. Pre-surgical treatment follows a standardized cadence (5 sessions/week for 3 weeks, then 3 sessions/week) for a minimum of 4 weeks, continuing until surgery or up to 10 weeks. Feasibility will be assessed using a pre-specified tiered exposure framework based on the number of sessions completed before surgery.

**Anticipated results:**

Primary outcomes are feasibility and acceptability of preoperative home-based tDCS delivery. The principal exploratory clinical outcome is change in depressive symptom severity measured using MADRS-S. Exploratory perioperative recovery outcomes include spine-related disability, pain intensity, functional status, and opioid utilization. For participants who proceed to surgery, postoperative outcomes will be followed up to 12 months.

**Discussion:**

This pilot study will generate feasibility, acceptability, and preliminary signal data to inform the design of future randomized controlled trials of preoperative neuromodulation in surgical populations.

## Introduction

Depressive symptoms are common among patients presenting for elective lumbar spine surgery and represent an under-addressed determinant of perioperative recovery (Kim et al., 2017; Javeed et al., 2024; Xu et al., 2026; O’Callaghan et al., 2026). Across spine populations, a substantial proportion of surgical candidates report clinically meaningful depressive symptoms at the time of evaluation, frequently in the setting of chronic pain and functional limitation. Importantly, this burden is evident even among patients without a formal psychiatric diagnosis, underscoring the clinical relevance of subsyndromal depressive symptoms in surgical care.

Depression in spine surgery candidates has been consistently associated with higher pain intensity, greater disability, and worse health-related quality of life at baseline (Kim et al., 2017; Javeed et al., 2024; Xu et al., 2026; O’Callaghan et al., 2026). Postoperatively, depressive symptoms have been linked to lower patient satisfaction, delayed functional recovery, and less favorable patient-reported outcomes. Depression has also been associated with increased perioperative opioid utilization and a greater likelihood of persistent opioid use after surgery, suggesting that mood disturbance may influence pain coping behaviors and recovery trajectories. Collectively, these findings position depression not merely as a comorbidity but as a potentially modifiable factor that shapes surgical outcomes.

The perioperative period represents a distinct opportunity for intervention. In elective lumbar surgery, the interval between surgical decision-making and operative intervention provides a predictable window during which targeted prehabilitation strategies can be implemented. While physical prehabilitation has received increasing attention, systematic approaches to optimizing mental health before spine surgery remain limited. Interventions that are scalable, remotely deliverable, and easily integrated into existing care pathways are particularly well suited to this setting.

Transcranial direct current stimulation (tDCS) is a noninvasive neuromodulation technique with established relevance to major depressive disorder. Bifrontal tDCS targeting the dorsolateral prefrontal cortex modulates cortical excitability and frontolimbic networks implicated in mood regulation, cognitive control, and pain processing. As a non-pharmacologic intervention, tDCS avoids medication-related adverse effects and drug–drug interactions that may complicate perioperative care. Its safety profile is favorable, with adverse effects typically mild and transient (Woodham et al., 2025).

Recent advances in device design, digital guidance, and remote supervision have enabled reliable home-based delivery of tDCS (Ruffini et al., 2024; Cappon et al., 2022). Clinical trials of home-based platforms have demonstrated feasibility, high adherence, and reductions in depressive symptom severity in both randomized and open-label settings, including fully remote implementations (Woodham et al., 2025; Borrione et al., 2024; Oh et al., 2023; Woodham et al., 2022; Moshfeghinia et al., 2025). These data support tDCS as a scalable intervention that can be deployed outside traditional psychiatric clinics, making it an attractive candidate for preoperative use (Aktürk et al., 2025).

The convergence of these clinical and technological developments makes elective lumbar spine surgery candidates with MDD a particularly well-suited population for home-based preoperative tDCS. First, the prevalence of clinically meaningful depressive symptoms is high in this cohort, and depression has a documented impact on perioperative outcomes including pain, function, and opioid utilization. Second, the elective surgical workflow provides a predictable, time-bounded preoperative window during which a structured intervention can be initiated and sustained. Third, home-based delivery is particularly relevant to spine surgery candidates, who often experience pain and functional limitations that may make repeated in-clinic visits burdensome. Fourth, the non-pharmacologic nature of tDCS avoids medication-related concerns that complicate perioperative pharmacotherapy decisions. Finally, the structured surgical care pathway provides a natural infrastructure for downstream outcome capture, enabling pragmatic evaluation of whether preoperative mood modulation is associated with recovery-relevant endpoints. Conceptually, reducing depressive symptom burden before surgery may improve pain coping, decrease pain interference, reduce opioid requirements, and support more favorable functional recovery. Despite this strong conceptual fit, the feasibility and acceptability of implementing home-based tDCS within the preoperative spine surgery pathway, and the extent to which sufficient treatment exposure can be achieved before surgery, remain unknown.

Before pursuing a definitive randomized controlled trial, foundational pilot data are needed. Specifically, it is necessary to determine whether preoperative home-based tDCS can be operationalized within the logistical constraints of elective spine surgery, whether patients can initiate and sustain treatment prior to surgery, and to obtain preliminary estimates of symptom change and variability. The present study addresses these gaps by evaluating the feasibility, acceptability, and preliminary clinical signal of preoperative home-based tDCS in elective, non-revision lumbar spine surgery candidates, informing the design of future controlled trials.

### Objectives and hypotheses

**Figure 1:**
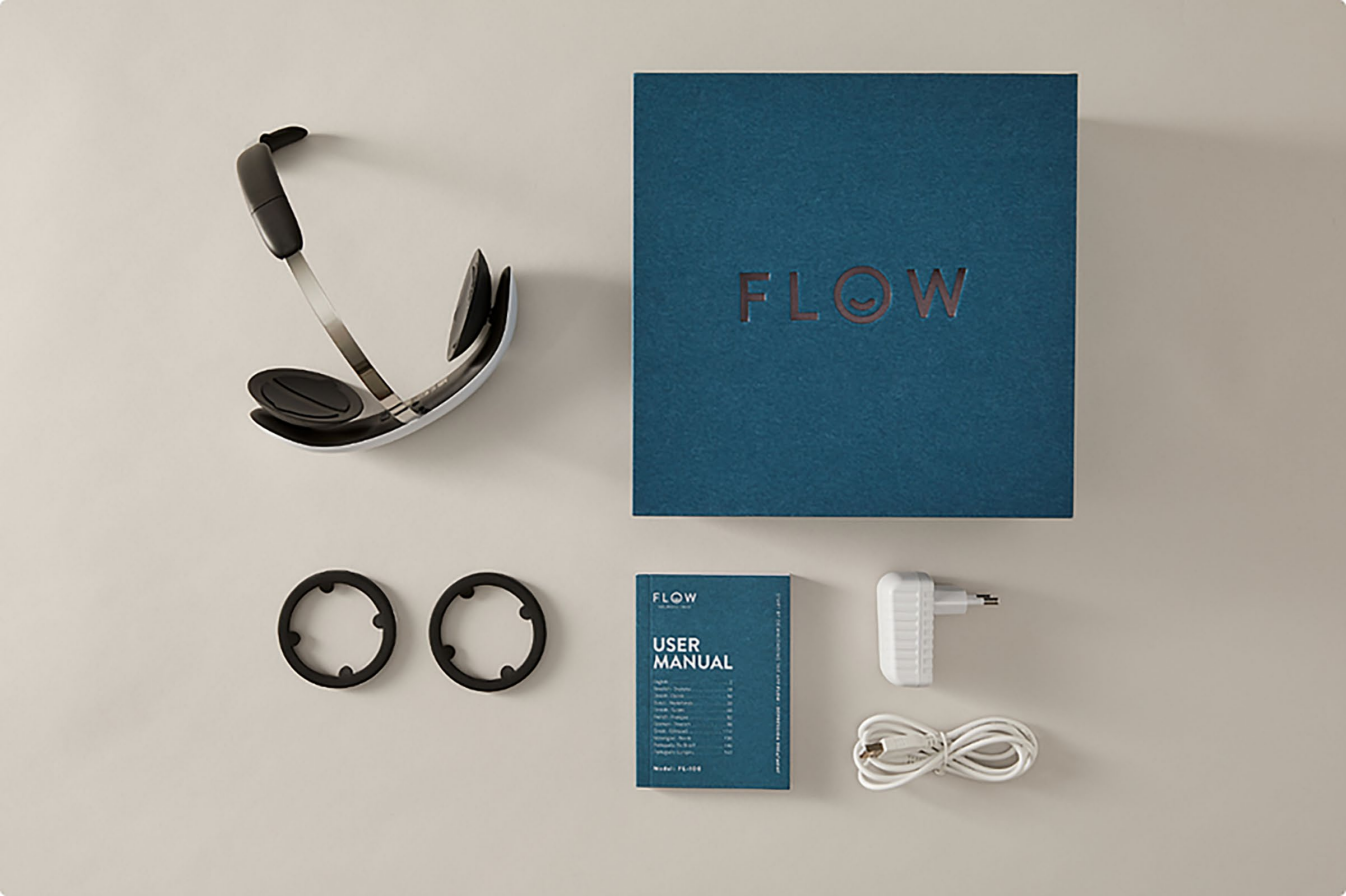
Flow neuroscience headset (FL-100) (Flow neuroscience patient sign-up, 2026). Source: Images were reproduced, with permission, from the parent company - Flow Neuroscience.

**Figure 2:**
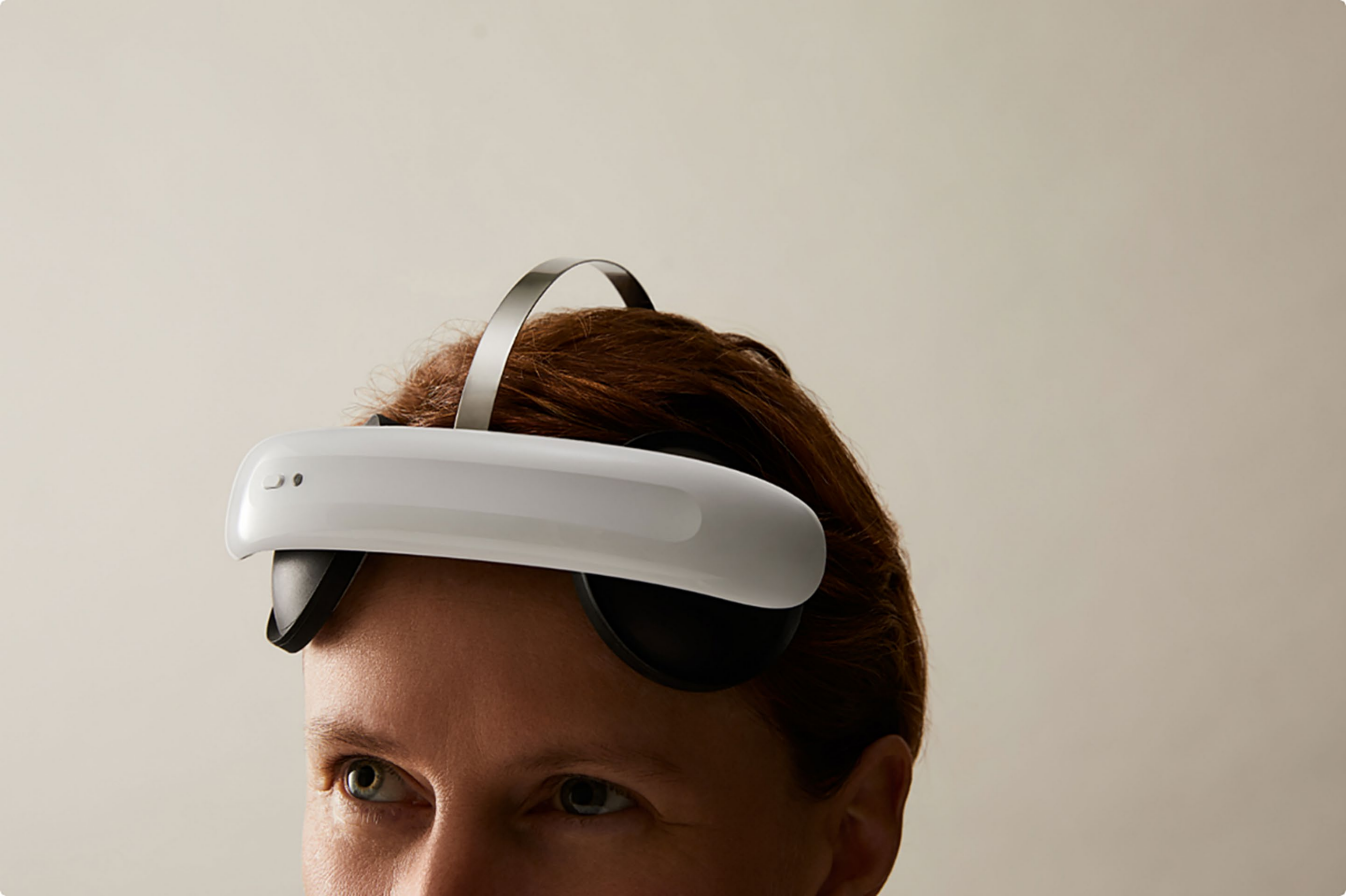
User wearing the flow neuroscience headset (Flow neuroscience patient sign-up, 2026). Source: Images were reproduced, with permission, from the parent company - Flow Neuroscience.

The primary objective of this study is to evaluate the feasibility and acceptability of delivering a home-based transcranial direct current stimulation (tDCS) intervention using the Flow Neuroscience device (Figures 1, 2) to patients with major depressive disorder (MDD) being evaluated for elective, non-revision lumbar spine surgery. The study aims to determine whether patients can successfully initiate and sustain a structured tDCS regimen during the preoperative period, despite variability in surgical timing and competing perioperative demands.

We hypothesize that most enrolled participants will be able to initiate treatment, defined as completion of the tDCS initiation phase within the first 3 weeks, and that a substantial proportion will achieve at least the minimum pre-specified treatment exposure before surgery. We further hypothesize that the intervention will be acceptable and well-tolerated, as reflected by high adherence, low rates of discontinuation due to adverse effects, and favorable participant-reported satisfaction.

A key exploratory clinical objective of the study is to assess preliminary changes in depressive symptom severity associated with preoperative home-based tDCS. The principal exploratory clinical outcome is change in depressive symptoms, measured using the self-reported Montgomery–Åsberg Depression Rating Scale (MADRS-S), consistent with prior home-based tDCS studies. We hypothesize that participants will demonstrate a reduction in depressive symptom severity from baseline to the preoperative assessment, with measurable improvement evident following completion of the intensive initiation phase.

Additional exploratory objectives include examining whether preoperative tDCS exposure is associated with changes in perioperative recovery-relevant outcomes. These exploratory outcomes include spine-related disability measured using the Oswestry Disability Index (ODI); back and leg pain intensity assessed via numeric rating scales; pain-related function measured using the PROMIS Pain Interference short form; and overall physical function measured using the PROMIS Physical Function short form. Opioid exposure will be assessed using standardized morphine milligram equivalent (MME) calculations, including baseline outpatient opioid use and postoperative opioid utilization across inpatient and outpatient settings.

In exploratory analyses, we hypothesize that greater tDCS exposure will be associated with larger reductions in depressive symptom severity and that participants demonstrating greater improvement in depressive symptoms may also exhibit lower opioid requirements and modest improvements in spine-related patient-reported outcomes. These analyses are intended to be hypothesis-generating and to inform outcome selection and effect size estimation for future controlled trials evaluating preoperative neuromodulation interventions in spine surgery populations.

This study is not powered to establish efficacy for clinical or surgical outcomes. Rather, it is designed to generate feasibility, acceptability, and preliminary signal data to guide the design of subsequent randomized studies.

## Methods and analysis

### Study design

**Figure 3:**
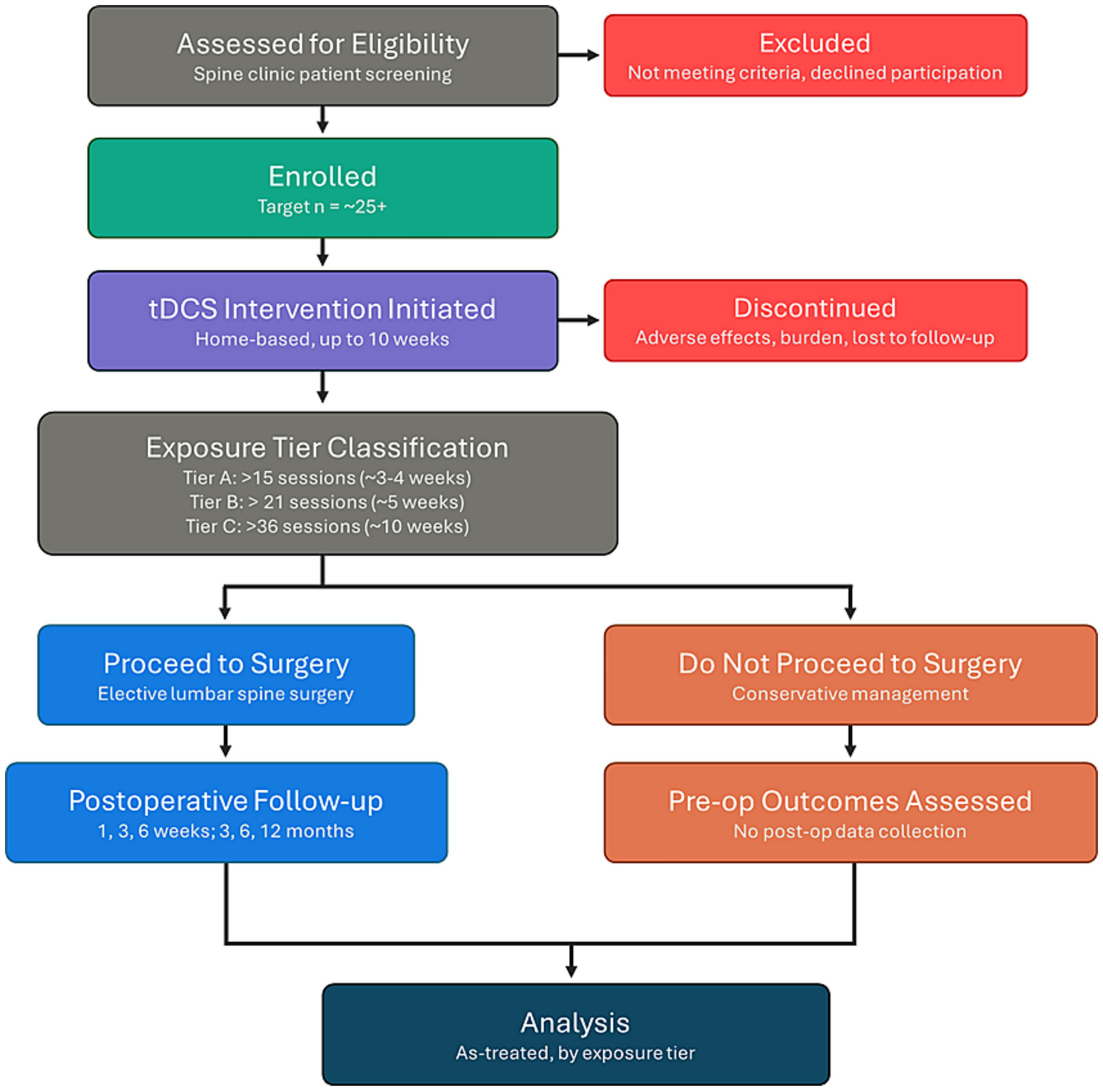
CONSORT-style participant flow diagram. Participants are identified through spine surgery clinic schedules and screened for eligibility. Enrolled participants initiate home-based tDCS and are classified by pre-specified exposure tier based on the number of sessions completed. Following the intervention period, participants who proceed to elective lumbar spine surgery enter postoperative follow-up. Participants who do not proceed to surgery are followed for pre-operative and intervention-related outcomes only; postoperative recovery endpoints are not collected for this subgroup. All participants are included in the primary as-treated analysis by exposure tier achieved. Source: Images were reproduced, with permission, from the parent company - Flow Neuroscience.

This is a prospective, open-label, single-arm feasibility study evaluating home-based transcranial direct current stimulation (tDCS) using the Flow Neuroscience device as a preoperative intervention for patients with major depressive disorder (MDD) being evaluated for elective, non-revision lumbar spine surgery (Figure 3).

The study is designed to prioritize feasibility and acceptability, with an exploratory assessment of changes in depressive symptom severity. Because the interval between surgical decision-making and operative intervention varies across patients, the intervention is delivered using a dose-to-time model with pre-specified exposure tiers based on the number of tDCS sessions completed before surgery.

As a pilot study, the design aims to (1) operationalize home-based neuromodulation within a perioperative spine surgery workflow, (2) quantify feasibility, acceptability, and safety, and (3) generate preliminary estimates of symptom change and variability to inform the design of future controlled trials. The protocol is reported in accordance with SPIRIT recommendations for interventional trial protocols (Supplementary Table S1).

### Setting

The study will be conducted at the Department of Neurosurgery and Department of Orthopaedic Surgery at Johns Hopkins Hospital, Baltimore, Maryland, United States. Enrollment is planned to begin in 2026, with completion of follow-up anticipated by 2027. Study procedures will be integrated with standard preoperative evaluation, perioperative care, and routine postoperative follow-up. The tDCS intervention is delivered in the home setting and supported through remote monitoring and scheduled study team check-ins. Study assessments may be completed in person or remotely, depending on clinical workflow and the timing of required measures.

### Eligibility criteria

Inclusion criteria:

1 Adults aged 22–75 years old
2 Clinical diagnosis of unipolar major depressive disorder as determined by the patient’s established managing clinician
3 At least moderate depressive symptom severity, defined as a baseline self-reported Montgomery–Åsberg Depression Rating Scale (MADRS-S) score ≥20
4 Being evaluated or scheduled for elective, non-revision lumbar spine surgery (prior spine surgery is not a contraindication)
5 Either:

- not receiving antidepressant medication, or
- receiving antidepressant medication and/or psychotherapy at a stable dose or frequency for ≥6 weeks before enrollment, with agreement to maintain treatment stability during the study

6 Ability to use the Flow device independently at home and to complete remote assessments, including use of a smartphone or tablet to guide sessions and transmit adherence data
7 Under the care of a primary care physician or mental health provider
8 Capacity to provide informed consent

Exclusion criteria: Exclusion criteria are designed to ensure participant safety, minimize risks associated with electrical stimulation, and improve interpretability of study outcomes. Key exclusions include:

Neurologic and device-related exclusions

- History of seizure disorder, epilepsy, loss of consciousness related to seizures, or other neurologic conditions that may increase risk
- Presence of implanted electronic or metallic devices (cranial or otherwise) that may pose safety concerns (e.g., pacemakers or implanted neurostimulators)
- Neurocranial defects

Psychiatric exclusions

- Clinically unstable psychiatric conditions requiring urgent treatment (e.g., acute suicidality, psychosis, or mania)
- Current or past bipolar disorder, psychotic disorder, or obsessive-compulsive disorder

tDCS-specific and medication exclusions

- Prior treatment with electroconvulsive therapy, transcranial magnetic stimulation, vagus nerve stimulation, or transcranial direct current stimulation*
  *Prior brain stimulation therapy is excluded to facilitate interpretation of feasibility and preliminary clinical signal in a neuromodulation-naïve population. Prior neuromodulation exposure may indicate treatment-resistant illness, alter cortical excitability, or introduce carryover effects that complicate interpretation of early-phase pilot outcomes. This criterion also aligns with the pivotal home-based tDCS trial of the Flow device to maintain consistency with the reference population in which efficacy was established.
- Did not complete 50% or more sessions
- Use of medications known to significantly affect cortical excitability (e.g., chronic benzodiazepines or anticonvulsant medications)
- Dermatologic contraindications at electrode sites (e.g., lesions or significant irritation)

Surgical exclusions

- Revision lumbar spine surgery
- Urgent or emergent surgical indications

Additional exclusions may include pregnancy, active opioid misuse or dependency, chronic pain conditions not primarily attributable to the operative pathology, or other conditions judged by the investigator to substantially increase risk or impede adherence to study procedures.

### Recruitment, screening, and informed consent

Potential participants will be identified by reviewing spine surgery clinic schedules. Eligible participants will complete written informed consent before any study-specific procedures. The consent process will emphasize voluntariness, the ability to withdraw at any time, and that participation will not affect standard surgical care. Surgical complexity will be quantified using validated procedure-based metrics derived from the operative record (e.g., surgical invasiveness/complexity indices and number of operative levels).

At enrollment, participants’ antidepressant treatment history will be systematically documented, including current and prior antidepressant medication trials (agent, dose, duration, and response), prior psychotherapy, and the number of failed adequate antidepressant trials in the current depressive episode. Treatment resistance status will not be used as an inclusion or exclusion criterion but will be reported descriptively to characterize the sample and support comparison with prior home-based tDCS studies that have enrolled treatment-resistant cohorts.

### Intervention: home-based tDCS regimen

Participants will receive a home-based tDCS intervention using a wearable headset (Flow FL-100 home tDCS headset (Buy or rent the flow headset—flow neuroscience, 2026) (Figures 1, 2) paired with a smartphone application that guides session delivery and logs adherence. Stimulation will be delivered at 2 mA for 30 min per session using a bilateral dorsolateral prefrontal cortex montage (anode over left DLPFC and cathode over right DLPFC), consistent with commonly used DLPFC-targeted protocols and the device’s pivotal trial (Woodham et al., 2025).

### Dosing schedule

All participants initiate treatment using a standardized dosing cadence derived from prior Flow Neuroscience trials. During the initiation phase (weeks 1–3), participants complete five tDCS sessions per week (15 sessions total). Beginning in week 4, participant’s transition to a maintenance phase of three sessions per week, continuing until the date of surgery or a maximum treatment duration of 10 weeks, whichever occurs first.

Because the interval between enrollment and surgery varies across participants, the total treatment duration is variable as well. The minimum planned exposure is 4 weeks, with a target exposure of 6–10 weeks when feasible. A treatment session is classified as complete if the full 30-min stimulation protocol is delivered, as recorded by the device, in accordance with protocol parameters. Sessions interrupted or terminated before 30 min will be classified as partial (≥20 min of stimulation delivered) or incomplete (<20 min of stimulation delivered). Only sessions classified as complete will count toward exposure tier classification. Partial and incomplete sessions will be documented and reported descriptively, with sensitivity analyses examining whether their inclusion materially affects feasibility metrics.

### Exposure tiers

To accommodate variability in surgical scheduling, participants are categorized based on the number of completed tDCS sessions before surgery:

- Tier A (initiation complete): ≥15 sessions (~3–4 weeks)
- Tier B (initiation + ≥ 1 maintenance week): ≥21 sessions (~5 weeks)
- Tier C (full exposure): 36 sessions (10-week regimen)

Participants will be analyzed on an as-treated basis, by the exposure tier achieved, regardless of intended treatment duration or if they proceeded with surgery.

### Onboarding and monitoring

Participants will receive standardized onboarding and device training, including in-clinic instruction when feasible and structured digital training materials. Training will emphasize correct headset placement and session initiation. Adherence will be monitored using application-generated usage logs, including session date, time, and completion status, which are accessible to the research team. Participants may be contacted weekly during the preoperative period to assess tolerability, address technical issues, review adherence, and screen for emerging concerns.

Deviations from the intended treatment schedule (e.g., missed sessions, delayed or rescheduled surgery dates) will be documented to support interpretation of feasibility outcomes and planned sensitivity analyses.

Tolerability will be explicitly assessed during onboarding sessions. Participants who experience intolerable adverse effects at the protocol-specified 2 mA intensity during initial onboarding (e.g., severe scalp discomfort, headache not relieved by standard measures, or other adverse effects leading to participant request to discontinue) will be documented as onboarding-phase discontinuations.

### Concomitant care

All participants will continue to receive standard surgical and perioperative care without modification. Concurrent treatment for depression, including pharmacotherapy and/or psychotherapy, will be permitted. Changes in depression-related treatment during the intervention and follow-up period—including medication initiation, discontinuation, or dose adjustment, as well as psychotherapy initiation—will be recorded systematically to support contextualization of outcome trajectories and exploratory sensitivity analyses.

### Follow-up schedule and study procedures

Participants will complete baseline assessments before initiation of tDCS (Supplementary Table S1—SPIRIT Schedule Breakdown). Depressive symptoms will be assessed longitudinally through the preoperative period, including interim assessments prior to surgery. Postoperative assessments will be collected at prespecified intervals, including early postoperative timepoints (2 and 6 weeks) and longer-term follow-up at 3, 6, and 12 months. Postoperative measures will focus on pain intensity, opioid utilization, functional outcomes, and health-related quality of life.

Participants who do not proceed to surgery will continue to be followed for preoperative and intervention-related outcomes, including depressive symptom trajectories, feasibility, and acceptability measures (Figure 3). However, endpoints that are specific to the postoperative recovery period will not be collected for these participants.

### Safety monitoring and stopping considerations

Expected risks of tDCS are primarily mild and transient, including headache, scalp discomfort, and skin irritation. Safety monitoring will occur throughout the intervention via participant self-report, scheduled study team check-ins, and systematic documentation of adverse events. Participants will be able to contact the research team at any time via a dedicated contact number. Adverse events (AEs) and serious adverse events (SAEs) will be recorded, assessed for severity and relatedness, and managed according to pre-specified procedures; tDCS-specific AEs will be assessed via the tDCS Adverse Events Questionnaire preoperatively (Brunoni et al., 2011). In the event of unanticipated device-related SAEs or emerging safety concerns, study procedures may be paused or halted in coordination with institutional oversight and applicable regulatory requirements.

A predefined mental health escalation pathway will be implemented for participants with clinically significant worsening of depressive symptoms or emergent suicidality. This pathway will include prompt clinical assessment and referral to appropriate mental health services when indicated (Appendix A).

### Data collection and management

Study data will be collected using secure electronic data capture tools [e.g., REDCap, (2026).] for participant-reported outcomes and study questionnaires, supplemented by abstraction of relevant clinical variables from the electronic health record as needed. Device and application-generated usage logs will be used to capture adherence and session completion metrics. Data will be stored in institutionally approved secure systems with access restricted to authorized study personnel. Data handling will follow applicable institutional policies and HIPAA requirements (Health Information Privacy, 2026). In addition, baseline pain and anxiety symptoms will be assessed using the Pain Catastrophizing Scale (PCS) and Generalized Anxiety Disorder-7 (GAD-7), respectively, to better characterize the cohort and support exploratory adjustment in analyses.

### Outcomes

#### Primary outcomes: feasibility and acceptability

The primary outcomes of this study are the feasibility and acceptability of delivering a preoperative, home-based tDCS intervention using the Flow device in elective lumbar spine surgery patients with MDD. Feasibility will be evaluated across two complementary domains: treatment initiation and overall treatment exposure. Feasibility of initiation will be defined as the proportion of enrolled participants who complete at least 15 tDCS sessions within the first 21 days, corresponding to successful completion of the intensive initiation phase. Feasibility of exposure will be assessed as the proportion of participants achieving each pre-specified exposure tier prior to surgery, reflecting the ability to sustain treatment over a variable preoperative timeframe.

Acceptability will be evaluated through structured measures of treatment satisfaction, usability, and tolerability, complemented by qualitative participant feedback. Treatment satisfaction will be assessed preoperatively using a structured questionnaire incorporating 5-point Likert items adapted from prior home-based tDCS feasibility work, evaluating overall acceptability (“very acceptable” to “very unacceptable”), perceived benefit, and willingness to recommend the intervention. Usability will be assessed using the System Usability Scale (SUS), a validated 10-item instrument yielding a normalized 0–100 score, administered preoperatively (Brooke, 1996). A brief semi-structured exit interview will be conducted at the preoperative timepoint to capture qualitative themes related to tolerability, integration into daily routines, and perceived burden. Tolerability will additionally be assessed by the frequency and nature of adverse effects, including those that lead to treatment interruption or discontinuation. Reasons for treatment discontinuation will be systematically recorded and categorized. Together, these outcomes are intended to characterize whether preoperative home-based tDCS can be.

#### Exploratory clinical outcomes: depressive symptoms

Clinical outcomes are exploratory (Table 1). The principal exploratory clinical outcome is change in depressive symptom severity measured using MADRS-S. Depressive symptoms will be assessed at baseline (week 0) before initiation of tDCS, at week 1, at the end of the initiation phase (week 4), at week 7, and at a preoperative assessment conducted within a defined window before surgery (week 8–10+). Observed changes in depressive symptom severity will be interpreted in the context of the study’s feasibility-focused design. These analyses are not intended to establish efficacy, but rather to generate preliminary signal and variability estimates to inform future trial design.

**Table 1:** Exploratory outcomes.

Domain	Measure	Baseline	Timepoints*	Pre-op**	Post-op
Depression	MADRS-S	X	X	X	—
Spine disability	Oswestry disability index (ODI)	X	—	X	X (6 wks or 3 mos)
Pain intensity	Back pain NRS (0–10)	X	X	X	X
	Leg pain NRS (0–10)	X	X	X	X
Pain-related function	PROMIS pain interference (SF)	X	Optional	X	X
Physical function	PROMIS physical function (SF)	X	—	X	X
Opioid exposure	Baseline outpatient opioid use (MME)	X	—	—	—
Opioid utilization	Inpatient postoperative MME	—	—	—	X
	30-day outpatient postoperative MME	—	—	—	X

*Week(s) 0, 1, 4, 7, 10, and preoperatively (as applicable). **≤7 days before surgery.

#### Exploratory clinical outcomes: perioperative recovery

Additional exploratory outcomes are included to examine whether preoperative home-based tDCS exposure is associated with changes in pain-related disability, functional status, and analgesic utilization in elective lumbar spine surgery candidates. Exploratory analyses will examine associations between tDCS exposure—both by pre-specified exposure tier and as a continuous measure of completed sessions—and changes in these outcomes.

Spine-specific disability will be assessed using ODI, administered at baseline, during the preoperative assessment window, and at a single exploratory postoperative time point. Pain intensity will be measured using 11-point numeric rating scales (NRS) for both back pain and leg pain. Pain-related functional impact will be assessed using the PROMIS Pain Interference short form, and overall physical function will be assessed using the PROMIS Physical Function short form.

Analgesic utilization will be evaluated using standardized morphine milligram equivalent (MME) calculations. Exploratory opioid outcomes will include baseline outpatient opioid exposure prior to initiation of tDCS, inpatient postoperative opioid utilization from surgery to hospital discharge, and cumulative outpatient opioid utilization during the early postoperative period.

#### Safety end points

Safety outcomes include the incidence and severity of adverse events (AEs) and serious adverse events (SAEs), as well as intervention discontinuations and protocol deviations attributable to tolerability or device use. Adverse events of interest include commonly reported minor effects such as scalp irritation, headache, and tinnitus. Safety outcomes will be summarized overall and, where relevant, by intervention phase.

### Analysis plan

Analyses will be conducted primarily to evaluate feasibility and acceptability, with clinical and perioperative outcomes analyzed on an exploratory, hypothesis-generating basis. All analyses will be prespecified and reported descriptively where appropriate, consistent with the pilot nature of the study.

#### Feasibility criteria

Feasibility will be evaluated across recruitment, initiation/adherence, acceptability, retention, and safety domains. The study will be considered feasible if (1) ≥ 60% of initiated participants achieve the minimum preoperative exposure target (Tier A, ≥15 total sessions completed before surgery); (2) overall retention is ≥50% for the preoperative assessment and ≥40% for the 30-day postoperative follow-up; (3) acceptability is supported by a mean treatment satisfaction/usability score ≥4/5 and ≤20% discontinuation attributable to device-related adverse effects or burden; and (4) safety is supported by no device-related serious adverse events, ≤10% escalation for emergent psychiatric concerns, and an adverse event profile consistent with expected transient tDCS effects (e.g., mild headache or scalp discomfort). Feasibility outcomes will be summarized descriptively with 95% confidence intervals and used to refine recruitment workflows, dosing schedules, and monitoring procedures for a subsequent randomized trial.

#### Analysis populations

The primary analytic population will include all enrolled participants who initiate the tDCS intervention. Participants will be analyzed on an as-treated basis, categorized according to the pre-specified exposure tier achieved before surgery. Sensitivity analyses may be performed using alternative adherence thresholds to assess the robustness of feasibility findings.

#### Sample size justification

The sample size is selected to provide reasonable precision around key feasibility metrics (treatment initiation, tier attainment, and adherence), and to generate preliminary estimates of variability in symptom change to inform a future randomized trial. With *n* = 20 participants who initiate tDCS, an observed feasibility proportion in the range expected for home-based neuromodulation (e.g., 60%–80%) would yield a 95% confidence interval width of ~0.34–0.40 (approximately ±0.17–0.20), which is adequate for feasibility decision-making and for informing go/no-go thresholds for subsequent trials. To account for anticipated attrition and scheduling-related drop-off before surgery, we will enroll *n* = 24–25 participants, targeting *n* = 20 evaluable participants who initiate treatment and contribute preoperative outcome assessments.

#### Primary analyses: feasibility and acceptability

Feasibility outcomes will be summarized using proportions and corresponding confidence intervals. These will include the proportion of participants who complete the initiation phase (≥15 sessions within 21 days) and the proportion achieving each pre-specified exposure tier before surgery. Adherence will be summarized as the proportion of prescribed sessions completed during the preoperative period. Acceptability outcomes will be summarized descriptively. System Usability Scale (SUS) scores will be reported as mean and standard deviation and classified against established benchmarks (a score >68 indicates above-average usability). Likert satisfaction items will be reported as proportions endorsing each response category. Qualitative exit-interview data will be analyzed using an established thematic-analysis framework to identify recurring themes in participant experience. Reasons for treatment discontinuation will be summarized descriptively.

#### Exploratory clinical analyses: depressive symptoms

Exploratory analyses will evaluate changes in depressive symptom severity over time. The principal exploratory clinical outcome is the change in MADRS-S score from baseline to the preoperative assessment. Change from baseline to the end of the initiation phase (week 4) will be examined as an early signal of response following intensive treatment initiation.

Longitudinal changes in depressive symptoms will be examined using mixed-effects models to account for repeated measures and variable treatment exposure. Models will include fixed effects for time and baseline symptom severity, with random effects to account for within-participant correlation. These analyses are exploratory and not powered to establish efficacy. Exploratory response and remission rates based on standard MADRS-S thresholds will be summarized descriptively at the preoperative time point.

#### Exploratory clinical analyses: perioperative recovery outcomes

Exploratory analyses will assess associations between preoperative tDCS exposure and perioperative recovery–related outcomes, including spine-related disability (ODI), pain intensity (back and leg NRS), pain interference (PROMIS Pain Interference), physical function (PROMIS Physical Function), and opioid utilization.

tDCS exposure will be modeled both categorically (by exposure tier) and continuously (as the number of completed sessions). Opioid utilization outcomes will be analyzed using standardized morphine milligram equivalents (MME), including baseline outpatient use, inpatient postoperative utilization, and cumulative outpatient use during the early postoperative period. Analyses will be descriptive and exploratory, with appropriate regression-based approaches used to examine associations while adjusting for baseline values where relevant.

#### Missing data

Missing data is anticipated due to the longitudinal design and perioperative context. Mixed-effects models inherently accommodate missing data under standard missing-at-random assumptions. The extent and pattern of missingness will be described, and additional sensitivity analyses may be considered if missingness is substantial.

#### Interim analyses and stopping rules

No formal interim efficacy analyses are planned. Safety will be monitored continuously throughout the study. Predefined safety considerations and institutional oversight requirements will guide decisions to pause or discontinue study procedures. Participant-level and study-level stopping will be pursued in the context of intolerable AE worsening depressive symptoms/emergent suicidality (defined in Appendix A), withdrawal of consent, pregnancy, or other safety flags deemed appropriate by the study team.

## Discussion

This prospective pilot study is designed to evaluate the feasibility and acceptability of delivering home-based transcranial direct current stimulation (tDCS) as a preoperative intervention elective lumbar spine surgery candidates with MDD. The primary contribution of this work is implementation-focused: to determine whether a remotely delivered neuromodulation intervention can be initiated and sustained within the variable and time-constrained preoperative surgical workflow. By using a dose-to-time framework with pre-specified exposure tiers, this study addresses a practical challenge unique to elective surgery—heterogeneity in time from enrollment to operation—and provides a pragmatic model for preoperative mental health optimization.

Beyond feasibility, this study aims to generate preliminary estimates of depressive symptom change and variability using a validated self-report instrument (MADRS-S). These exploratory clinical data are not designed to establish efficacy, but rather to inform endpoint selection, assessment timing, and sample size assumptions for future trials. In addition, exploratory evaluation of perioperative recovery–relevant outcomes, including pain, disability, and opioid utilization, provides an initial signal as to whether preoperative mood modulation may be meaningfully related to downstream recovery trajectories.

Several anticipated challenges are inherent to this study design. Adherence may vary over longer preoperative intervals, and treatment exposure will be constrained by surgical scheduling. Concomitant changes in depression treatment during the preoperative period may introduce additional variability. Surgical heterogeneity and baseline differences in pain and disability further complicate the interpretation of exploratory recovery outcomes. These challenges are intentionally addressed through feasibility-first endpoints, as-treated analyses, tiered exposure definitions, and descriptive modeling approaches, which prioritize transparency and interpretability over causal inference.

This study has important limitations. As an open-label, single-arm pilot, it is inherently susceptible to placebo and context effects, regression to the mean, and spontaneous symptom fluctuation. Selection bias is possible, as participants are limited to elective, non-revision lumbar spine surgery candidates who are able and willing to engage with a home-based digital intervention. Accordingly, findings may not generalize to emergent surgery, revision procedures, or other spine populations. These limitations are appropriate for an early-phase feasibility study and underscore the need for subsequent controlled evaluation.

The results of this study are intended to directly inform next steps. A logical extension is a randomized, sham-controlled or comparative effectiveness trial evaluating preoperative tDCS in surgical populations. Future work should also assess implementation outcomes, including scalability, cost-effectiveness, and integration into perioperative care pathways. Incorporating mechanistic or intermediate outcomes may further clarify links among mood modulation, pain processing, and recovery. Together, this pilot study represents a foundational step toward evaluating preoperative neuromodulation as a scalable strategy to optimize mental health and recovery in spine surgery.

## Ethics and dissemination

This study has received and will be conducted in accordance with Institutional Review Board approval (IRB00498020) and FDA nonsignificant risk classification (G210328). All participants will provide written informed consent before enrollment. Participant safety will be monitored throughout the intervention and follow-up periods, including structured assessment of adverse events and a predefined pathway for escalation of emergent mental health concerns.

Findings will be disseminated through presentations at scientific meetings and publications in peer-reviewed journals. Trial registration details, protocol versioning, and protocol amendments will be reported in accordance with the journal’s and registry’s expectations.
