# Supplementary material for: A prospective, open-label feasibility study protocol of home-based transcranial direct current stimulation for major depressive disorder in elective lumbar spine surgery candidates
Source: Front Hum Neurosci. 2026 Jun 11;20:1860063. doi: 10.3389/fnhum.2026.1860063 (PMC13294146; doi:10.3389/fnhum.2026.1860063)
Supplement: Supplementary file 1 [file Supplementary_file_1.DOCX]

**APPENDIX A**

**Mental Health Escalation Pathway for Worsening Depressive Symptoms and Suicidality**

**Purpose**

To ensure participant safety, a predefined mental health escalation pathway will be implemented for (1) clinically significant worsening of depressive symptoms and/or (2) emergent suicidality identified at any point during screening, intervention, or follow-up. This pathway is intended to support timely clinical assessment, appropriate referral, and emergency intervention when needed, while recognizing that study participation does not replace standard psychiatric care.

**Scope**

Applies to all participants from screening through completion of follow-up and may be activated based on information obtained through:

- Scheduled study assessments
- Preoperative check-ins
- Unscheduled participant contact with the study team
- Clinical team concerns observed during in-person encounters
- Reports from family members or caregivers (when applicable and permitted)

**Definitions and safety triggers**

The pathway will be triggered by any of the following, regardless of study phase:

**A. Worsening depressive symptoms (non-emergent trigger)**

Indicators of clinically meaningful worsening may include:

- Marked increase in depressive symptom scores compared with baseline or prior assessment
- Severe worsening of functional impairment attributable to mood symptoms
- Concerning reports of sustained worsening mood, anhedonia, hopelessness, or inability to complete daily activities
- Clinician concerns that symptoms exceed what is appropriate for continued unsupervised participation

**B. Emergent suicidality (urgent or emergent trigger)**

Emergent suicidality triggers include:

- Any endorsement of suicidal ideation with intent, plan, or preparatory behaviors (e.g., self-reported or clinician-detected)*
- Escalating suicidal ideation severity or new onset of suicidal ideation during the study period
- Recent self-harm behavior or suicide attempt
- Imminent concern for participant safety expressed by the participant or observed by the study team

*Note: Passive thoughts of death or self-harm screening item endorsement will prompt follow-up safety assessment to determine urgency and appropriate escalation.

**Escalation tiers & actions**

*Tier 1 - Mild-to-moderate worsening depressive symptoms (non-urgent)*

Criteria (examples): worsening mood symptoms without imminent safety concern; no suicidal intent/plan; participant remains able to engage in care.

Actions:

- Clinical check-in: The study clinician (or designee) will contact the participant to clarify symptom trajectory and assess clinical stability.
- Psychiatry referral: A non-urgent psychiatry consultation will be made (telehealth or in-person, based on availability and participant preference), and/or the participant will be encouraged to contact their established mental health clinician if already in care.
- Study continuity decision: Participants may continue the intervention with enhanced monitoring if clinically appropriate.

*Tier 2 - Significant worsening or concerning symptoms (urgent)*

Criteria (examples): substantial symptom escalation; new suicidal ideation without clear intent/plan; inability to ensure safe home participation; clinician concern for rapid deterioration.

Actions:

- Same-day clinical assessment: A same-day telephone or video assessment will be conducted by the study clinician or psychiatry service when feasible.
- Urgent psychiatry consultation: An urgent psychiatry consultation will be initiated if indicated.
- Temporary pause of study intervention: The tDCS intervention may be paused pending clinical evaluation, at the discretion of the study clinician/PI.
- Care coordination: The participant’s existing clinicians (e.g., primary care or treating psychiatrist, when applicable and permitted) may be notified to support continuity and safety.
- Safety planning: A safety plan and clear instructions for accessing urgent care will be discussed and documented, consistent with institutional standards.

*Tier 3 - Emergent suicidality / imminent risk (emergency)*

Criteria: suicidal ideation with intent/plan; recent attempt; inability to ensure safety; imminent risk identified during contact or observed in-person.

Actions:

- Immediate emergency response: The study team will initiate immediate escalation consistent with institutional policy. Participants will be instructed to seek emergency evaluation through the Emergency Department (ED) or emergency services when clinically indicated.
  - If the participant is in-person: The participant will not be left alone, and institutional emergency protocols will be activated to facilitate urgent evaluation. If clinically indicated, a pathway to maintain safety during transfer and evaluation (including a supervised hold) will be implemented in accordance with institutional policy and applicable law.
  - If the participant is remote: The study clinician will remain on the line when possible, facilitate a connection to emergency services, and—if imminent risk is suspected and safety cannot be confirmed—may request a welfare check or emergency response, consistent with institutional practice and participant safety obligations.
- Study intervention discontinuation/pause: The tDCS intervention will be stopped or paused until the participant is clinically stabilized and cleared for continued participation.

**Post-escalation follow-up and return-to-study considerations**

Following Tier 2 or Tier 3 escalation, the study team will reassess whether continued participation is appropriate. Factors informing return-to-study include:

- Clinical stabilization and absence of imminent safety risk
- Psychiatry recommendations (if consulted)
- Participant willingness and capacity to continue study procedures
- Ability to safely complete a home-based protocol

If continued participation is not appropriate, the participant may be withdrawn for safety, and withdrawal will not affect clinical care.

**Documentation and reporting**

All escalations will be documented in the study record, including: trigger event(s), time of identification, assessment performed, actions taken, and referrals initiated.

**APPENDIX B**

**Supplementary Table 1.** SPIRIT Schedule Breakdown

| **Study procedure** | **Enrollment** | **Pre-operative intervention period** | | | | | **Post-operative follow-up^†^** | | | | |
| --- | --- | --- | --- | --- | --- | --- | --- | --- | --- | --- | --- |
|  | **Screen** | **Wk 0** | **Wk 1** | **Wk 4** | **Wk 7** | **Pre-op*** | **2 wk** | **6 wk** | **3 mo** | **6 mo** | **12 mo** |
| ***Enrollment*** | | | | | | | | | | | |
| Eligibility screen & Informed Consent | X |  |  |  |  |  |  |  |  |  |  |
| MDD evaluation via MADRS-S | X |  |  |  |  |  |  |  |  |  |  |
| Demographics & medical history | X |  |  |  |  |  |  |  |  |  |  |
| ***Interventions*** | | | | | | | | | | | |
| tDCS initiation phase (5×/wk) |  |  |  |  |  |  |  |  |  |  |  |
| tDCS maintenance phase (3×/wk) |  |  |  |  |  |  | Opt | Opt | Opt | Opt | Opt |
| Device onboarding & training |  | X |  |  |  |  |  |  |  |  |  |
| ***Assessments — depression*** | | | | | | | | | | | |
| MADRS-S (self-report) |  | X | X | X | X | X |  |  |  |  |  |
| GAD-7 |  | X |  |  |  |  |  |  |  |  |  |
| ***Assessments — spine & pain*** | | | | | | | | | | | |
| Oswestry Disability Index (ODI) |  | X | X | X | X | X |  | X | X |  |  |
| Back pain NRS (0–10) |  | X | X | X | X | X | X | X |  |  |  |
| Leg pain NRS (0–10) |  | X | X | X | X | X | X | X |  |  |  |
| PROMIS Pain Interference (SF) |  | X | Opt |  |  | X |  | X | X | X | X |
| PROMIS Physical Function (SF) |  | X |  |  |  | X |  | X | X | X | X |
| Pain Catastrophizing Scale (PCS) |  | X |  |  |  |  |  |  |  |  |  |
| ***Assessments — opioid utilization*** | | | | | | | | | | | |
| Baseline outpatient opioid use (MME) |  | X |  |  |  |  |  |  |  |  |  |
| Postoperative MME |  |  |  |  |  |  | X | X |  |  |  |
| ***Safety & feasibility*** | | | | | | | | | | | |
| Adverse event monitoring |  |  |  |  |  |  |  |  |  |  |  |
| tDCS Adverse Events Questionnaire |  |  |  |  |  | X |  |  |  |  |  |
| Treatment satisfaction / usability |  |  |  |  |  | X |  |  |  |  |  |
| Concomitant medication changes |  |  |  |  |  |  |  |  |  |  |  |

*Or end of treatment phase if surgery is not pursued or delayed (as applicable)

**^†^**Post-operative assessments apply only to participants who proceed to surgery; participants managed conservatively will be followed for pre-operative and intervention-related outcomes only.

“X” denotes data collection at the specified timepoint

Shaded blue cells indicate continuous intervention period

Shaded purple cells indicate ongoing monitoring throughout the study.

Pre-op assessment is conducted within 7 days before surgery

opt = optional collection
